# Comparison of biogenic silver nanoparticles formed by *Momordica charantia* and *Psidium guajava* leaf extract and antifungal evaluation

**DOI:** 10.1371/journal.pone.0239360

**Published:** 2020-09-22

**Authors:** Dai Hai Nguyen, Thanh Nguyet Nguyen Vo, Ngoc Tung Nguyen, Yern Chee Ching, Thai Thanh Hoang Thi

**Affiliations:** 1 Institute of Applied Materials Science, Vietnam Academy of Science and Technology, Ho Chi Minh City, Vietnam; 2 Graduate University of Science and Technology, Vietnam Academy of Science and Technology, Hanoi, Vietnam; 3 Biomaterials and Nanotechnology Research Group, Faculty of Applied Sciences, Ton Duc Thang University, Ho Chi Minh City, Vietnam; 4 Center for Research and Technology Transfer, Vietnam Academy of Science and Technology, Hanoi, Vietnam; 5 Department of Chemical Engineering, Faculty of Engineering, University of Malaya, Kuala Lumpur, Malaysia; National University of Ireland, Galway, IRELAND

## Abstract

Exploiting plant extracts to form metallic nanoparticles has been becoming the promising alternative routes of chemical and physical methods owing to environmentally friendly and abundantly renewable resources. In this study, *Momordica charantia* and *Psidium guajava* leaf extract (MC.broth and PG.broth) are exploited to fabricate two kinds of biogenic silver nanoparticles (MC.AgNPs and PG.AgNPs). Phytoconstituent screening is performed to identify the categories of natural compounds in MC.broth and PG.broth. Both extracts contain wealthy polyphenols which play a role of reducing agent to turn silver (I) ions into silver nuclei. Trace alkaloids, rich saponins and other oxygen-containing compounds creating the organic corona surrounding nanoparticles act as stabilizing agents. MC.AgNPs and PG.AgNPs are characterized by UV-vis and FTIR spectrophotometry, EDS and TEM techniques. FTIR spectra indicate the presence of O-H, C = O, C-O-C and C = C groups on the surface of silver nanoparticles which is corresponded with three elements of C, O and Ag found in EDS analysis. TEM micrographs show the spherical morphology of MC.AgNPs and PG.AgNPs. MC.AgNPs were 17.0 nm distributed in narrow range of 5–29 nm, while the average size of PG.AgNPs were 25.7 nm in the range of 5–53 nm. Further, MC.AgNPs and PG.AgNPs exhibit their effectively inhibitory ability against *A*. *niger*, *A*. *flavus* and *F*. *oxysporum* as dose-dependence. Altogether, MC.AgNPs and PG.AgNPs will have much potential in scaled up production and become the promising fungicides for agricultural applications.

## Introduction

The development of nanotechnology has designed various nanoparticles with multi-function for many applications such as photovoltaic devices, catalysts, sensors and biomedical/pharmaceutical/antimicrobial products [1]. Metallic nanoparticles have gained high attention because of their unique characteristics including surface plasmon resonance, thermo-optical properties and inherently antimicrobial capacity [2]. Depending on particular needs, the nanoparticle properties were tailored differently. The green synthesis of metallic nanoparticles have been becoming the alternative routes of chemical and physical methods owing to lower costs, environmentally friendly and abundantly renewable resources [3–5]. Among green methods, using plant extracts to fabricate silver nanoparticles (AgNPs) has gained much attention because of fast procedure, simplicity and feasibility of scaling-up production. Especially, it does not need to maintain the complex conditions for microbial culture of the AgNP synthesis utilizing fungi, bacteria and other organisms [6]. In addition, plant biodiversity has opened various topics for worldwide researchers. To obtain the improved AgNPs with the desired and controllable properties, a myriad of tropical/herbal plants could be studied [7]. By exploiting this route, the biogenic AgNPs were able to be formed within the range less than 100 nm. These sizes facilitate AgNPs to go through the bacteria/fungi walls and damage membranes as well as to release Ag^+^ to disturb some mechanisms in cells [7, 8]. In addition, phytochemicals not only could stabilize AgNPs but also act as an effective molecules in antimicrobial activities [7].

From 1900s, the biogenic AgNPs fabricated by plant extracts have been reported [9]. The general mechanism was mainly explained by the dual function of secondary metabolites which could reduce silver ions and stabilize AgNPs in one-pot reaction [9]. Till date, various plant species were exploited to fabricate green AgNPs and explore their physicochemical properties and bioactivities. For example, to obtain the green AgNPs with diameter less than 30 nm, it could be utilized the extracts of banana peel, *Boerhaavia diffusa*, olive leaf, *Prunus japonica* leaf, dried grass, pomegranate juice, *Taraxacum officinale* leaf, *Acalypha indica* leaf, *Juglans regia* bark, *Cucumis sativus* [10], *Acacia leucophloea* bark, *Adiantum philippense* fronds or *Bacopa monneria* [9]. In contrary, the bigger green AgNPs (more than 100 nm) were formed by *Ficus carica* latex, *Euphorbia lacteal*, *Euphorbia milii* latex, *Euphorbia ingens* latex, *Euphorbia hirta* leaf, *Eucalyptus hybrid* leaf, *Citrus limon* and *Azadirachta indica* leaf [9], *Caltropis procera* fruit and leaf, *Rheum palmatum* root [10]. About nanoparticle shape, these plant extract-mediated AgNPs were impacted by plant species, it could be spherical, cubic, hexagonal or multi-shaped morphology [9–11]. The size and shape of AgNPs were indicated to be significantly impacted by reducing power and stabilizers of reaction mixture. These reagents effected on the growing and agglomerating stages of AgNP formation. Thus the different components in each extract were considered to vary the reducing and capping ability to fabricate green AgNPs that would assist to finely tune the physicochemical properties of green AgNPs. Herein, *Momordica charantia* and *Psidium guajava* were chosen.

*Momordica charantia* (*M*. *charantia*) belongs to the Cucurbitaceae family [12]. Possessing a rich source of bioactive compounds, *M*. *charantia* exhibited the remarkably biological and pharmacological activities in anti-cancer, anti-inflammation, anti-diabetes, anti-hyperglycemia, anti-oxidant and hepatoprotection [12]. The recent studies on active compounds of *M*. *charantia* reported the main ingredients were polysaccharides, proteins, saponins, phenolic compounds (catechin, caffeic acid and gallic acid) [13–15]. *Psidium guajava (P*. *guajava)* belongs to the Myrtaceae family. *P*. *guajava* possesses many medicinal activities [16]. It was proved that *P*. *guajava* had positive effects on hepatoprotection, anti-inflammation, anti-oxidant, anti-diarrhea, anti-stomachache, anti-cancer, antioxidant, anti-bacteria, anti-hyperglycemia and antispasmodic [16]. The main components of *P*. *guajava* were found to contain flavonoids, vitamins, phenolic compounds, tannins, essential oils, triterpenoid acids, carotenoids and sesquiterpene [16–18]. Although all parts of the tree were able to use for nanoparticle synthesis, only leaf was chosen to fabricate AgNPs because it contains abundantly bioactive ingredients. In addition, leaf harvest is easy, leaf regeneration is rapid that will bring an economical effect.

In this study, the green methods biosynthesizing AgNPs were performed with the aqueous extracts of *M*. *charantia* and *P*. *guajava* leaves (MC.broth and PG.broth). Phytoconstituent screening was carried out to recognize the main phytochemical categories as well as to estimate qualitatively the difference between two these extracts. The biogenic silver nanoparticles (MC.AgNPs and PG.AgNPs) fabricated by MC.broth and PG.broth were characterized by UV-vis and FTIR spectrophotometry, EDS and TEM techniques. Furthermore, the formation reactions and stabilizing mechanism were suggested based on phytochemical compositions and the physicochemical properties of these green AgNPs. In order to demonstrate the antifungal activities of MC.AgNPs and PG.AgNPs, three fungi including *Aspergillus niger*, *Fusarium oxysporum* and *Aspergillus flavus* were cultured on the agar dishes containing different concentrations of these biogenic AgNPs.

## Materials and methods

### Materials

*Momordica charantia* and *Psidium guajava* leaves were supplied by the medicinal plant garden of Tra Vinh University (Tra Vinh province, Vietnam). Silver nitrate (AgNO_3_), sulfuric acid (H_2_SO_4_), ferric chloride (FeCl_3_), potassium iodide (KI), potassium bromide (KBr) and iodine (I_2_) were bought from Sigma-Aldrich (Merck, Darmstadt, Germany). Acetic anhydride ((CH_3_CO)_2_O) was purchased from Labkem (Casablanca, Morocco, US). *F*. *oxysporum*, *A*. *niger* and *A*. *flavus* were taken from Institute of Applied Materials Science, Vietnam Academy of Science and Technology (Ho Chi Minh city, Vietnam). Potato dextrose agar (PDA, composition: agar of 15 g/L, dextrose of 20 g/L, potato extract of 4 g/L) was bought from Sigma-Aldrich (Merck, Darmstadt, Germany). Deionized water (DIW) was purified by Milli-Q HX 7150 machine (Merck Millipore, France).

#### Plant broth preparation

**Fig 1:**
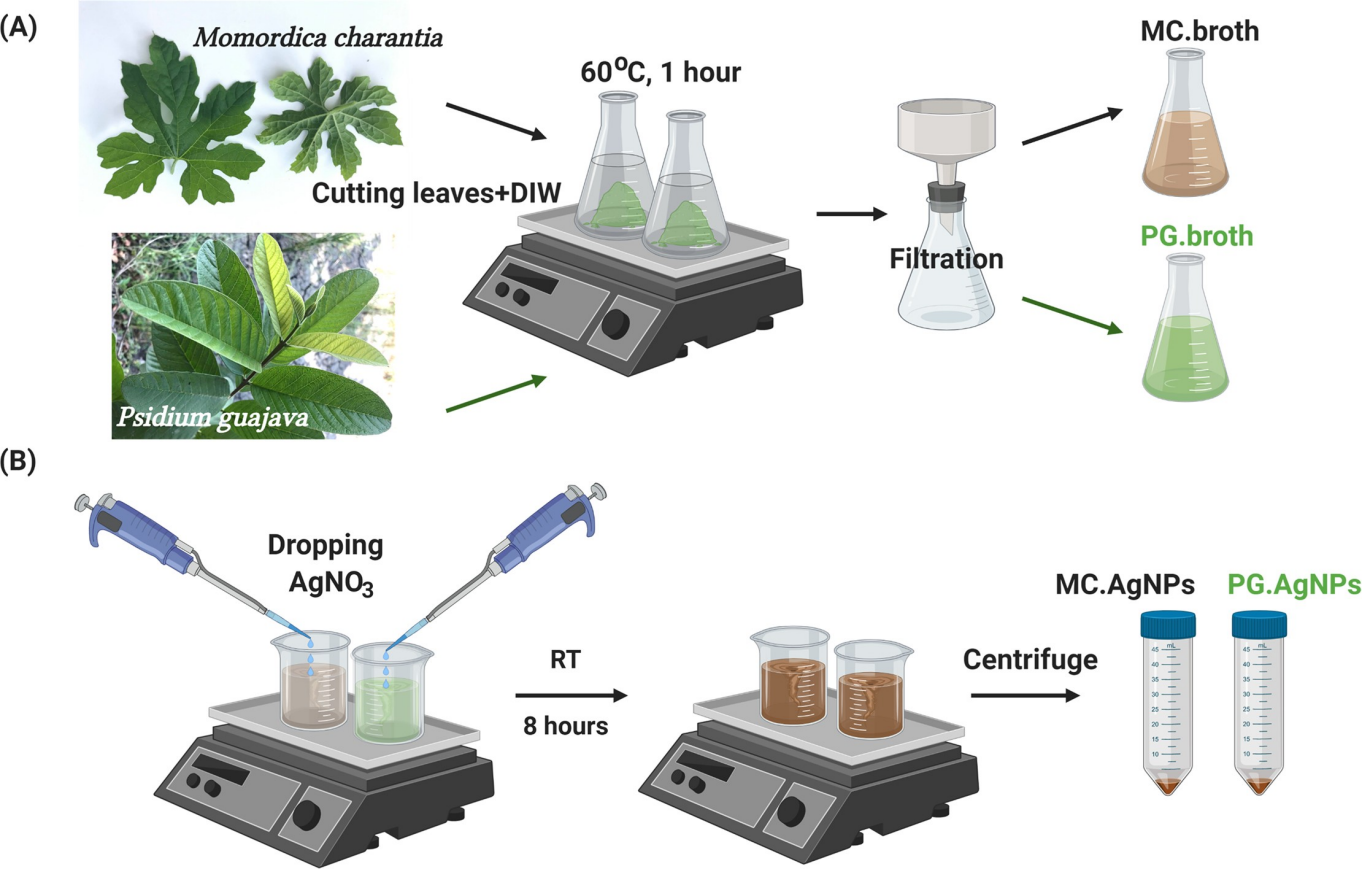
Extraction procedure of aqueous *Momordica charantia* and *Psidium guajava* leaf extracts (respectively symbolized as MC.broth and PG.broth) (A); Biosynthesis procedure of silver nanoparticles using MC.broth and PG.broth (respectively symbolized as MC.AgNPs and PG.AgNPs). (Created with BioRender.com).

*Momordica charantia* and *Psidium guajava* leaves without diseases and contamination were carefully chosen. After washing with deionized water three times, each type of leaves was dried at 60°C in oven. After chopping, *Momordica charantia* and *Psidium guajava* leaves (5 g) were added into two different erlenmeyer flasks. DIW of 100 mL was added into each flask. These flasks were heated to 60°C for 1 hour. Then these aqueous extracts of *Momordica charantia* and *Psidium guajava* leaves were filtered by porcelain Buchner filter funnels lined by two Whatman No.1 filter paper sheets, being symbolized as MC.broth and PG.broth respectively (Fig 1A). These broths were poured in brown bottles stored at 4°C for other experiments.

### Phytoconstituent screening

To test alkaloids, Wagner’s reagent was utilized. Three mL of extract was put into test tube. Three mL of concentrated H_2_SO_4_ was added into extract. Wagner’s reagent (2.5 g I_2_ in 250 mL KI solution (5 wt%)) was dropped into an acidified extract. If alkaloids were present, the red-brown precipitate was observed.

To qualitatively analyze saponins, the frothing test was performed. Five mL of extract was put into test tube. One test tube containing MC.broth and another tube containing PG.broth were shaken at the same time for few minutes. The presence of saponins was confirmed if the appeared froth was stable more than 10 minutes.

To identify phenolics and tannins, FeCl_3_ test was used. The extract (3 mL) was added into test tube followed by dropping FeCl_3_ (5 wt%) of 1 mL. A bluish-black color was produced [19] that could confirm the presence of tannins and phenolics.

To test steroids and triterpenes, Liebermann Burchard test was carried out. Three mL of extract was mixed with acetic anhydride (few drops) in the test tube. One mL of concentrated H_2_SO_4_ was added. The steroid was present when there was an appeared green color. If the pink color was seen, there was a presence of triterpenoids.

### Green synthesis of AgNPs using *Momordica charantia* and *Psidium guajava* leaf extracts

Aqueous solution 1 mM of silver nitrate was prepared for the AgNP biosynthesis. Two beakers contained 4.5 mL of silver nitrate. Then 0.5 mL of MC.broth and PG.broth was separately added into these silver nitrate beakers. These mixtures were stirred for 8 hours at room temperature (RT). The AgNPs fabricated by MC.broth and PG.broth were named as MC.AgNPs and PG.AgNPs respectively. After completing reaction, MC.AgNPs and PG.AgNPs were centrifuged to collect the suspension in the bottom of tube. Adding DIW for washing, then the centrifugation was performed to obtain MC.AgNPs and PG.AgNPs. The washing step was repeated 3 times. The MC.AgNPs and PG.AgNPs were stored in vacuum conditions after lyophilized.

### Characterization of MC.AgNPs and PG.AgNPs

The MC.broth, PG.broth, and two reacted mixtures of MC.AgNPs and PG.AgNPs after 8 hours were fivefold diluted with DIW to collect their UV-vis spectra using UV-vis spectrometer (Shimadzu UV-1800, US). The UV-vis spectra were scanned from 350 to 750 nm.

The lyophilized MC.broth, PG.broth, MC.AgNPs and PG.AgNPs were respectively crushed with KBr at the weight ratio of 1:10. These samples were pelleted and analyzed by FTIR spectrophotometer (Perkin Elmer, US). The scanning wavenumber was in the range of 4000–500 cm^-1^.

The size and morphology of MC.AgNPs and PG.AgNPs were observed by TEM (JEOL model JEM-1400, Japan). The elemental composition of MC.AgNPs and PG.AgNPs was confirmed with EDS (Horiba H-7593, UK).

### Antifungal activity of MC.AgNPs and PG.AgNPs

PDA (39 g) was dissolved in DIW (1 L). PDA solution was sterilized at 121 ^o^C for 15 minutes. The sterilized PDA solution was poured into the Petri dishes and cooled to form the agar dishes which were shortly called PDA dishes. The sterilized PDA solution was cooled to around 55 ^o^C to mix with tested agents including 0.5 mL of MC.broth, 0.5 mL of PG.broth, MC.AgNPs (20 and 40 ppm) and PG.AgNPs (20 and 40 ppm). The mixtures of PDA solution and each tested agent were poured directly into Petri dishes. The code of each agar dish was explained in Table 1.

**Table 1:** The code of agar dishes utilized in antifungal tests.

Agar dish code	Compositions of agar dishes
PDA	Sterilized PDA solutions
MC.broth	Sterilized PDA solution + 0.5 mL of MC.broth
PG.broth	Sterilized PDA solution + 0.5 mL of PG.broth
MC.AgNP20	MC.AgNPs homogenized in a sterilized PDA solution at 20 ppm
MC.AgNP40	MC.AgNPs homogenized in a sterilized PDA solution at 40 ppm
PG.AgNP20	PG.AgNPs homogenized in a sterilized PDA solution at 20 ppm
PG.AgNP40	PG.AgNPs homogenized in a sterilized PDA solution at 40 ppm

Three pathogenic organisms including *Aspergillus niger*, *Fusarium oxysporum*, *Aspergillus flavus* were utilized for antifungal tests of MC.AgNPs and PG.AgNPs. Each fungal strain was spotted in the center of each dish. Each experiment was replicated three times. The incubation temperature was 30°C. After 24, 48, 72, and 96 hours, the diameters of fungal zones were recorded and averaged. The values were presented as mean ± standard deviation (SD).

### Statistical analysis

The experiments were replicated three times and represented as mean ± standard deviation. Student’s *t* test was utilized to analyze all experimental data. *P* < 0.05 implied that two compared results were statistically significant. *P* > 0.05 indicated non-statistical difference.

## Results and discussion

### Qualitative tests of phytoconstituents’ MC.broth and PG.broth

**Fig 2:**
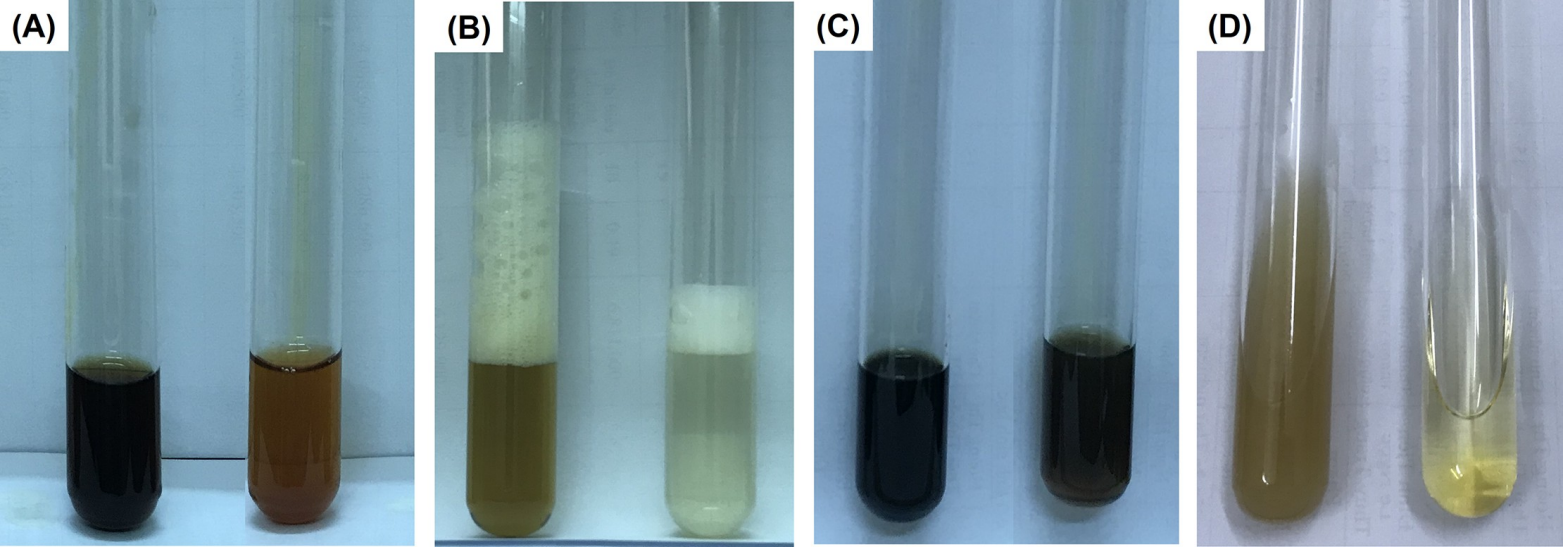
The Wagner’s test: adding solution of I_2_/KI into MC.broth (left) and PG.broth (right), an occurrence of very little red-brown precipitate in MC.broth indicated tracing alkaloid presence (A); foam test: after shaking MC.broth (left) and PG.broth (right) test tubes for few minutes, the froth formation being stable for 10 minutes showed the presence of saponins (B); FeCl_3_ test: after FeCl_3_ was respectively added into MC.broth (left) and PG.broth (right), the color was turned into bluish-black color that implied the presence of polyphenols (C); Liebermann Burchard test: MC.broth (left) and PG.broth (right) were tested by Liebermann Burchard reaction, green or pink color was not appeared that implied the absence of steroids and triterpenes (D).

In aqueous extracts of MC.broth and PG.broth, the phytoconstituent screening tests revealed the presence of some secondary metabolites (Fig 2 and Table 2). Very small amount of red-brown precipitation was observed in MC.broth, but not occurred in PG.broth (Fig 2A) that confirm the trace alkaloids in MC.broth. Fig 2B showed stable froth in both MC.broth and PG.broth, the height of foam layer observed in MC.broth was significantly higher than that in PG.broth. This phenomenon could estimate the saponin amount in MC.broth being 2.5-fold higher than saponin amount in PG.broth. The MC.broth and PG.broth had bluish dark color with high intensity when adding FeCl_3_ that implied the abundant presence of tannin and polyphenols (Fig 2C). Indeed, many researches confirmed that the phenolics and saponins (e.g. gallic acid, *p-*coumaric acid, chlorogenic acid, tannic acid, rutin, naringin, quercetin, epicatechin(-), genistein, naringenin and daidzein) distributed in various *M*. *charantia* tissues including leaves [20–22]. Diaz-de-Cerio et al. studied about polar compounds in guava leaves, they found 13 ellagic and gallic acid derivatives, quercetin and its derivatives, catechin, gallocatechin, gallic acid, naringenin and many guavinosides [17]. In case of Liebermann Burchard test, no reaction was happened that demonstrated an absence of steroids and triterpenes in MC.broth and PG.broth. Through phytochemical screening results, it was realized that the MC.broth contained more compound categories and especially richer saponins than PG.broth (Table 2).

**Table 2:** The phytoconstituent screening of MC.broth and PG.broth.

Tests	MC.broth	PG.broth	Phytoconstituents
**Wagner’s test**	+	-	Alkaloids
**Foam test**	+++++	++	Saponins
**FeCl**_**3**_ **test**	+++	+++	Tannins and phenolics
**Liebermann Burchard test**	-	-	Steroids and triterpenes

Notes: (-): absence; (+) trace amount; (++), (+++), (+++++): presence at low, medium and high level.

### Synthesis of MC.AgNPs and PG.AgNPs

**Fig 3:**
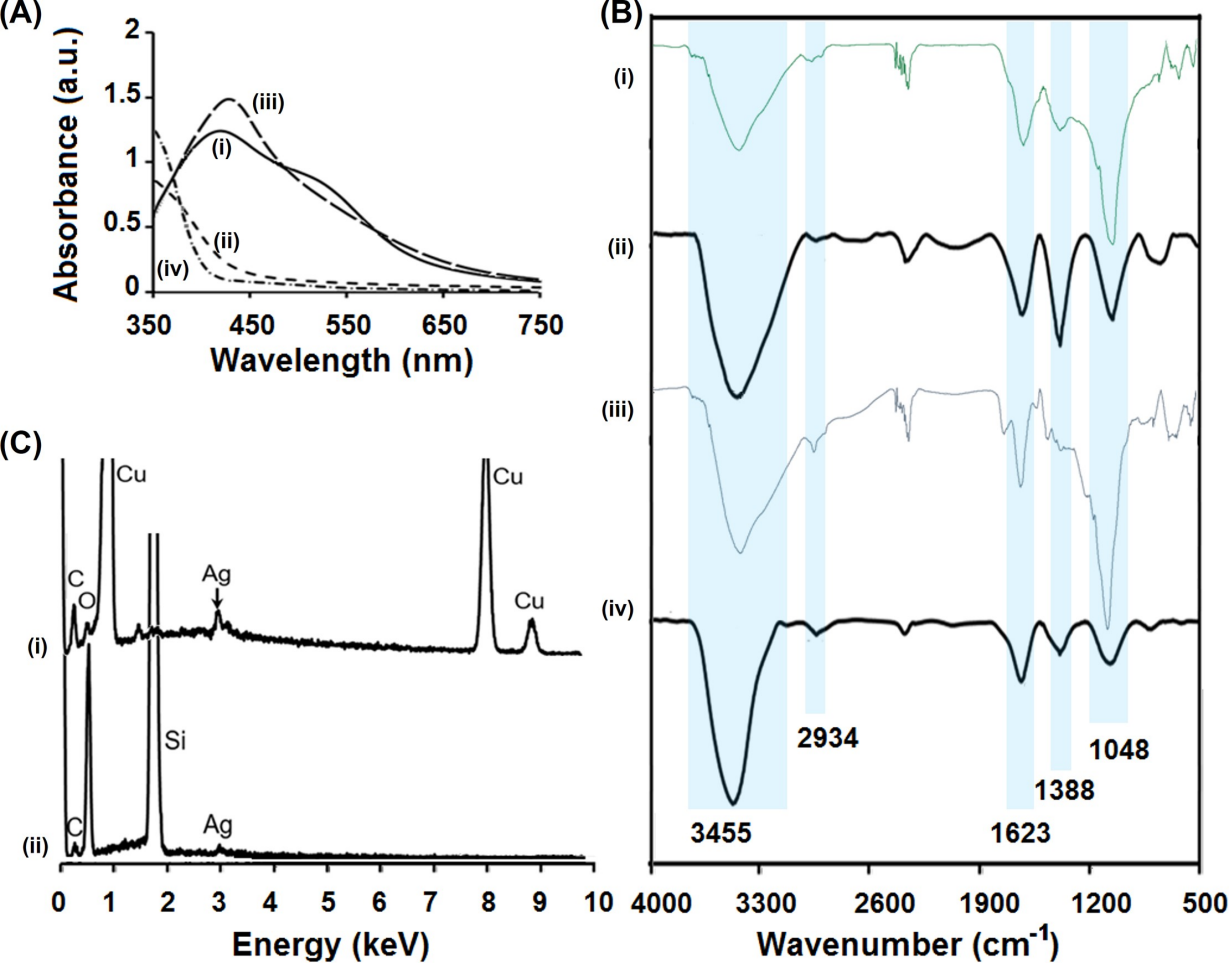
UV-vis spectra of MC.AgNPs (i), MC.broth (ii), PG.AgNPs (iii) and PG.broth (iv): The absorption peak of 420 nm was appeared in both MC.AgNPs and PG.AgNPs that indicated the presence of silver nanoparticles (A); FTIR spectra of MC.broth (i), MC.AgNPs (ii), PG.broth (iii) and PG.AgNPs (iv) showed the presence of organic functional groups (B); EDS spectra of MC.AgNPs (i) and PG.AgNPs (ii) exhibited that silver, carbon and oxygen were contained in MC.AgNPs and PG.AgNPs, while Si and Cu were strange elements contaminated by TEM grids (C).

The MC.broth had brown color while PG.broth showed light yellow color. After dropping Ag^+^ solution and reacting for 8 hours at room temperature (RT), the color of two mixtures turned reddish brown. This color change indicated the AgNP formation. In MC.broth and PG.broth, many phenolic compounds were present. Phenolic compounds theirselves could play a role of reducing agents to reduce Ag^+^ into Ag^o^ [7, 23]. Then Ag nuclei were coagulated to grow into silver nanoparticles. Depending on the stabilizing agents (saponins, alkaloids and oxidized compounds), silver nanoparticles were defined their stability, dimension and morphology. To demonstrate the formation of silver nanoparticles, UV-Vis spectrophotometry was utilized. Due to the local surface plasmon resonance (SPR), AgNPs could absorb the light in UV-vis region. For clear observation, two reacted mixtures and two extracts were scanned by UV-Vis spectrophotometer from 350 to 750 nm. The Fig 3 showed the UV-vis spectra of MC.AgNPs (Fig 3A-i) and PG.AgNPs (Fig 3A-iii) in comparison with MC.broth (Fig 3A-ii) and PG.broth (Fig 3A-iv). The absorption peak of 420 nm was appeared in both MC.AgNPs and PG.AgNPs that indicated the occurrence of AgNPs [24], while two spectra of extracts didn’t show that peak. Besides, the MC.AgNPs exhibited another broaden peaks at 540 nm tangled with the 420-nm peak that implied the aggregation behavior. Owing to the higher phytoconstituents of MC.broth, the organic compounds were attached on the surface of MC.AgNPs more than PG.AgNPs, that induced the secondary aggregate due to the interaction of nanoparticle organic-shells [25].

The FTIR spectrophotometer was operated to identify the organic functional groups on the surface of two AgNP types (MC.AgNPs and PG.AgNPs) in comparison with two extracts (MC.broth and PG.broth) (Fig 3B). In all spectra, it was observed the same main troughs of 3455, 2934, 1623, 1388 and 1048 cm^-1^ respectively assigned to–OH stretching,–CH stretching,–C = O or–C = C–stretching,–CH_3_ bending,–C–O–C–stretching vibrations. The FTIR results revealed that the functional groups of MC.AgNPs and PG.AgNPs were similar together and corresponded with MC.broth and PG.broth. From this fact, it was inferred that the phytoconstituents of extracts attached on the surface of their biosynthesized AgNPs. However, all peaks of each FTIR spectra in the fingerprint regions (500–1500 cm^-1^) were not the same that showed the difference in compound structures of MC.broth, MC.AgNPs, PG.broth and PG.AgNPs. From FTIR results, considering about element components, it was also realized that the MC.AgNPs and PG.AgNPs contained C and O. The EDS analysis was applied to confirm this conclusion as well as demonstrate the presence of Ag. As predicted, the EDS spectra of MC.AgNPs and PG.AgNPs (Fig 3C-i and 3C-ii) indicated the presence of C, O and Ag. The strange occurrence of Cu and Si was explained by contaminating from the grid holder [4, 26]. Taken together, the formation mechanism of MC.AgNPs and PG.AgNPs was suggested that the natural compounds belong to phenolics have mainly four types such as phenol, catechol, meta-benzenediol and pyrogallol groups which could donate electron to silver (I) ions to generate silver nuclei. In the reacting mixture, residue of unreacted phenolics, oxidizing forms of phenolics after reaction, saponins and others containing oxygen which can bind with AgNP surface through coordination bonds.

### The size and morphology of MC.AgNPs and PG.AgNPs

**Fig 4:**
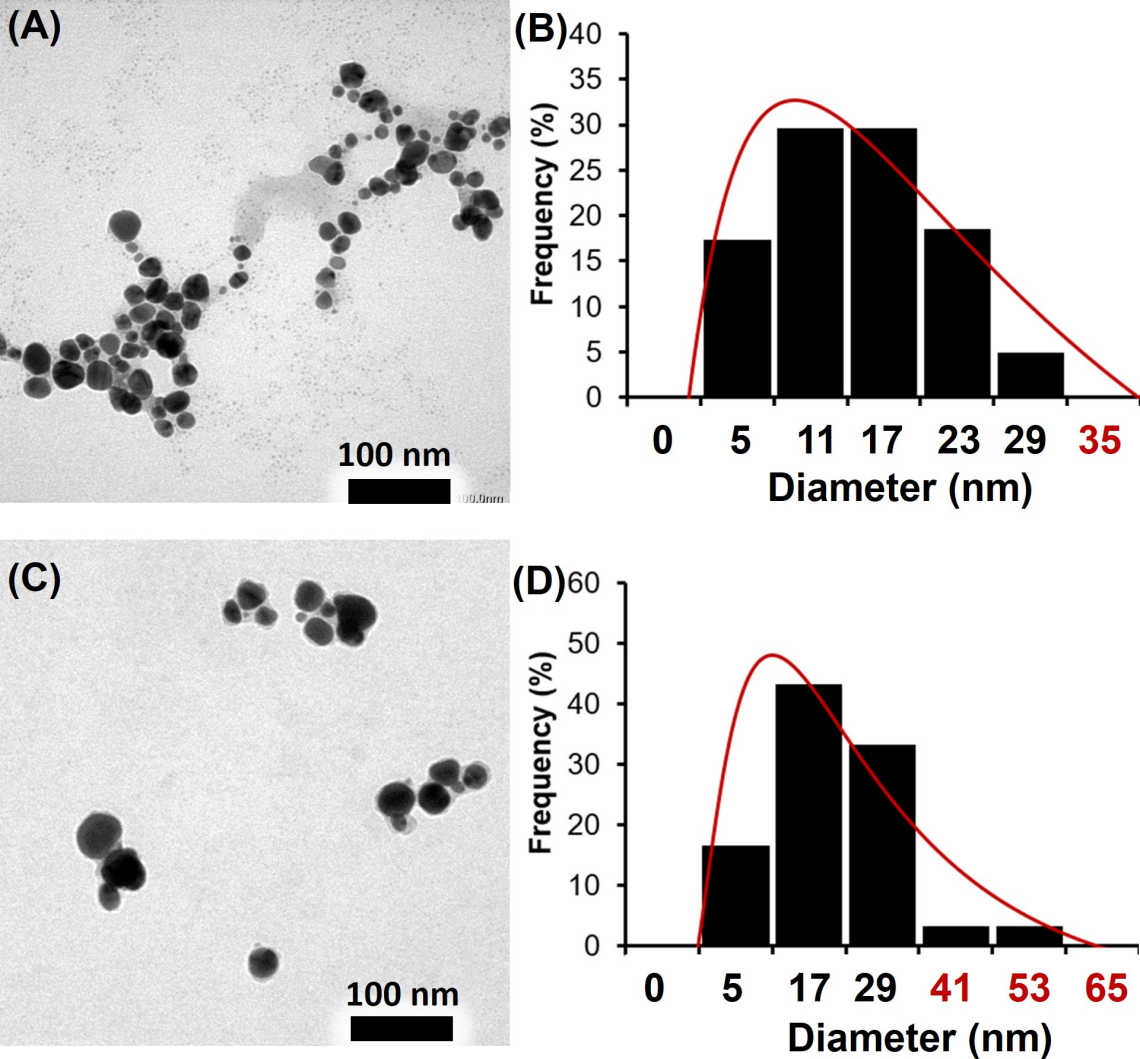
The MC.AgNPs’ TEM micrograph (A) showed the spherical shape and individual nanoparticles, but some MC.AgNPs trapped in the blurred membrane; the size distribution graph of MC.AgNPs (B) exhibited that MC.AgNPs’dimension was in the range of 5–29 nm; the PG.AgNPs’ TEM micrograph (C) implied that the PG.AgNPs were less cluster than MC.AgNPs; the size distribution graph of PG.AgNPs (D) indicated that PG.AgNPs were in the range of 5–53 nm.

The morphology and particle size of MC.AgNPs and PG.AgNPs were examined by TEM technique. The MC.AgNPs (Fig 4A) were almost spherical shape and distributed separately together, but some MC.AgNPs trapped in the blurred membrane were observed as clusters. This TEM image of MC.AgNPs was agreed with their above UV-vis spectrum showing the secondary aggregation. Fig 4B exhibited the graph of MC.AgNPs’ size distribution which were in the range of 5–29 nm with the average diameter of 17.0 nm. The TEM micrographs of PG.AgNPs (Fig 4C) showed their spherical morphology. Also the blurred membrane kept some PG.AgNPs together but the number of PG.AgNPs in cluster was less than MC.AgNPs. Fig 4D exhibited the PG.AgNP size range of 5–53 nm, the average diameter was 25.7 nm. Making the comparison, the MC.AgNPs were in narrower size distribution and smaller diameter than PG.AgNPs. Specically, MC.AgNPs had over 75% of small nanoparticles being less than or equal 17 nm which PG.AgNPs achieved only 60%. Other 25% of MC.AgNPs was 23 and 29 nm, while 40% of PG.AgNPs was 29, 41 and 53 nm. Considering the phytoconstituent screening results, it was realized MC.broth with the presence of alkaloids and richer saponin than PG.broth that leads to create the smaller MC.AgNPs than PG.AgNPs. The explanation might be due to high content of capping agents (saponins, alkaloids and other phytoconstituents) inhibiting the silver seed growth into bigger AgNPs. Taken together, all MC.AgNPs and PG.AgNPs were obtained with the nano-size less than 53 nm. This dimension could completely meet the requirements for antimicrobial applications which need the nanoparticles being less than 100 nm. At this scale, the nanoparticles can penetrate into the microorganism membranes to inhibit their growth through interacting with many kinds of proteins, DNA and enzymes [7, 8].

### Antifungal activity of MC.AgNPs and PG.AgNPs

To examine the antifungal activity of MC.AgNPs and PG.AgNPs, three fungal strains including *A*. *niger*, *A*. *flavus* and *F*. *oxysporum* were seeded in the center of PDA agar dishes in which MC.broth, PG.broth, MC.AgNPs and PG.AgNPs were fortified at various amounts. The mycelium zones of each dish were measured every 24 hours (Table 3). The fungal diameter on each dish was compared to that of PDA dishes, the inhibition taken place when the mycelium zones was smaller than PDA media.

**Table 3:** The diameter of mycelium zones (mm) of *A*. *niger*, *A*. *flavus* and *F*. *oxysporum* proliferated on various agar dishes.

		PDA	MC.broth	MC.AgNP20	MC.AgNP40	PG.broth	PG.AgNP20	PG.AgNP40
***A*. *niger***	**24h**	24.5 ± 0.5	24.8 ± 0.2	18.8 ± 0.2	14.6 ± 0.5	24.5 ± 0.2	19.3 ± 0.5	12.8 ± 0.2
	**48h**	44.6 ± 0.5	44.3 ± 1.1	28.5 ± 0.8	22.3 ± 1.1	44.8 ± 0.5	30.0 ± 0.5	18.8 ± 0.7
	**72h**	65.1 ± 0.2	64.8 ± 0.2	45.8 ± 0.7	36.8 ± 0.2	64.2 ± 0.5	46.8 ± 0.2	34.1 ± 0.2
	**96h**	89.5 ± 0.5	88.6 ± 1.1	66.5 ± 0.5	53.3 ± 0.5	89.5 ± 0.5	67.1 ± 0.2	48.3 ± 0.5
***A*. *flavus***	**24h**	20.6 ± 0.2	20.3 ± 0.5	16.8 ± 0.2	12.8 ± 0.2	20.7 ± 0.2	16.5 ± 0.5	11.8 ± 0.2
	**48h**	32.8 ± 0.2	31.0 ± 1.0	24.0 ± 0.5	18.0 ± 0.5	32.3 ± 0.8	24.8 ± 0.2	16.5 ± 0.5
	**72h**	57.5 ± 0.5	56.0 ± 1.0	41.1 ± 0.2	33.1 ± 0.2	54.7 ± 0.5	43.5 ± 0.5	27.8 ± 0.2
	**96h**	89.3 ± 1.1	88.3 ± 1.5	60.1 ± 0.2	48.8 ± 0.2	88.8 ± 0.2	61.3 ± 0.2	43.1 ± 0.2
***F*. *oxysporum***	**24h**	11.1 ± 0.2	11.0 ± 0.8	7.8 ± 0.2	7.3 ± 0.2	10.9 ± 0.2	7.3 ± 0.2	5.8 ± 0.5
	**48h**	24.0 ± 0.5	23.5 ± 0.5	17.8 ± 0.2	12.1 ± 1.0	24.5 ± 0.8	16.0 ± 1.0	11.0 ± 1.5
	**72h**	52.0 ± 1.0	50.1 ± 0.7	35.7 ± 0.8	22.0 ± 1.0	52.0 ± 0.5	31.6 ± 0.7	16.5 ± 0.5
	**96h**	73.3 ± 1.5	71.3 ± 1.5	49.1 ± 1.2	30.6 ± 0.5	72.8 ± 0.2	43.5 ± 0.5	23.1 ± 0.2

On PDA dishes (control sample), after 24, 48, 72 and 96 hours, the *A*. *niger* mycelium diameters respectively were 24.5, 44.6, 65.1 and 89.5 mm; the *A*. *flavus* diameters were 20.6, 32.8, 57.5 and 89.3 mm; the *F*. *oxysporum* ones were 11.1, 24.0, 52.0 and 73.3 mm. After 96 hours, *A*. *niger* and *A*. *flavus* were spread out full surface of dishes, but *F*. *oxysporum* achieved only near edge of dishes. So *F*. *oxysporum* proliferation was slower than two others (*P* < 0.05). On MC.broth, at the time points of 24-hour, 48-hour, 72-hour and 96-hour incubation, *A*. *niger* diameters were 24.8, 44.3, 64.8 and 88.6 mm; *A*. *flavus* diameters were 20.3, 31.0, 56.0 and 88.3 mm; *F*. *oxysporum* diameters were 11.0, 23.5, 50.1 and 71.3 mm. On PG.broth, with these respectively above time intervals, *A*. *niger* diameters were 24.5, 44.8, 64.2 and 89.5 mm; *A*. *flavus* diameters were 20.7, 32.3, 54.7 and 88.8 mm; *F*. *oxysporum* diameters were 10.9, 24.5, 52.0 and 72.8 mm. Making the comparison between two extracts with PDA dishes, the radial growth of three fungal strains were similar together at each time interval (*P* > 0.05). MC.broth and PG.broth didn’t exhibit the antifungal effect against *A*. *niger*, *A*. *flavus* and *F*. *oxysporum* at the using concentration for synthesis of AgNPs. Being similar to other studies [4, 26], the diluted concentration of leaf extracts was not enough strong activity to inhibit the fungal growth.

In case of MC.AgNP20 and PG.AgNP20, as presented detail in Table 3, the *A*. *niger*, *A*. *flavus* and *F*. *oxysporum* zones were significantly smaller than that on PDA dishes (*P* < 0.05). For example, after 96-hour incubation, the mycelium zones on MC.AgNP20 were 66.5, 60.1 and 49.1 mm; and the mycelium zones on PG.AgNP20 were 67.1, 61.3 and 43.5 mm for *A*. *niger*, *A*. *flavus* and *F*. *oxysporum* respectively (Table 3). The similarity of fungal diameters on MC.AgNP20 and PG.AgNP20 (*P* >0.05) implied that two these green AgNPs achieved the same antifungal ability. It was explained by their nanosize being less than 100 nm which dimension was easily able to penetrate inside fungal cells. This penetration of AgNPs could cause the wounds or leakages on fungal membranes. In the biological environment, AgNPs interacted with oxidizers to sustainably generate silver (I) ions. Then Ag^+^ ions could strongly bind with proteins, enzymes and DNA of fungi to break their bio processes that leads to fungus death [27]. Besides, AgNPs were also effectively anchored on the cell membranes or inside fungal cells to disturb the metabolism or proliferation. When increasing the green AgNP concentration to 40 ppm, the radial growth of *A*. *niger* and *A*. *flavus* was decreased one fourth-fold, *F*. *oxysporum* was a half fold deduction. As a result, MC.AgNPs and PG.AgNPs could show the effective antifungal ability in dose-dependence. Looking at Kim et al.’s study, they utilized the commercial AgNPs to test with 18 different fungal strains [28]. The commercial AgNPs exhibited antifungal properties at various concentrations of 10, 25, 50 and 100 ppm [28]. About dose as well as inhibitory effect, the MC.AgNPs and PG.AgNPs were corresponded with those commercial AgNPs. In case of an antimicrobial mechanism, silver nanoparticles were degraded in biologically reducing media to release Ag^+^ ions interacting the proteins of cell membranes or inside cell walls that led to inhibit cell division or cause cell dead [8]. Thus these MC.AgNPs and PG.AgNPs covered with naturally organic functional groups might achieve the long-term biodegradation as well as the sustainable release of Ag^+^ ions *in vivo*. As a result, MC.AgNPs and PG.AgNPs might exhibit the long-term antimicrobial activities. Indeed, surface coating of nanoparticles has been become the strategy for controlling the biodegradation of metallic nanoparticles in physiological conditions. Polysaccharides and poly(ethylene glycol) could modify the metallic nanoparticle surface to prolong their half-life *in vivo* [8, 29]. However, poly(ethylene glycol)-modified nanoparticles become more vulnerable than bare ones both *in vitro* and *in vivo* [29] because the generation of anti-polymer antibodies caused the accelerated clearance for poly(ethylene glycol)-modified nanoparticles [30]. The same situation also occurred for other synthetic polymers [30], so they should be not considered in surface modification. Taken together, the natural compounds outside MC.AgNPs and PG.AgNPs might become a promising strategy to stabilize silver nanoparticles for long-term activities and avoid the anti-polymer antibody generation.

Nowadays, metallic nanoparticles have been applied prevalently in consumer products, agriculture, medical and high-tech fields that induced the worrisome consequence of nanoparticle pollution. However, silver has been considered as a metal possessing least toxicity even in the accumulation state [31]. In spite of that, the rapid development of silver nanoparticles led emerging concerns related to nano-sized silver toxicity on ecosystem and humans [32]. In case of using AgNPs for agricultural applications, AgNPs will accumulated on soil and/or in sludge that might exhibit the bioactivities in next crops [33]. However, the transport and fate of AgNPs are complicated to fully understand [32, 33], it might be leaked into water. In addition, the useful species might be influenced by silver nanoparticles, also the Ag^+^ resistance might happen. Thus the aquatic environment must be controlled to achieve the silver content in acceptable level.

## Conclusion

In summary, both aqueous *Momordica charantia* and *Psidium guajava* leaf extracts could be successfully applied to fabricated green AgNPs. MC.broth possessed richer phytoconstituents, especially trace alkaloids and more wealthy saponins, than PG.broth that led to form two types of AgNPs being different in dimension and size distribution. UV-vis spectra of MC.AgNPs revealed that the two entangled peaks of 420 nm and 540 nm indicated the formation of AgNPs with secondary aggregation due to physical interaction of organic corona, while PG.AgNPs had only a sharp peak centered at 420 nm. By using TEM technique, the spherical morphology of MC.AgNPs and PG.AgNPs were observed. MC.AgNPs were 17.0 nm distributed in narrow range of 5–29 nm, while PG.AgNPs were 25.7 nm in the nanoscale from 5 to 53 nm. FTIR and EDS spectra confirmed both two these AgNPs were capped with the functional groups originated from leaf extracts. Thus MC.AgNPs and PG.AgNPs were able to be stabilized without any additional steps. Due to their size less than 100 nm, MC.AgNPs and PG.AgNPs could show their highly antifungal efficiency against *A*. *niger*, *A*. *flavus* and *F*. *oxysporum*. So these green AgNPs were synthesized by ecofriendly method overcoming the disadvantages of traditional ones. In the future, MC.AgNPs and PG.AgNPs could be scaled up production and become the promising fungicides for protection of crops.

## References

[1] Kim H.; Park Y.; Stevens M.M.; Kwon W.; Hahn S.K. Multifunctional hyaluronate—nanoparticle hybrid systems for diagnostic, therapeutic and theranostic applications. Journal of controlled release: official journal of the Controlled Release Society 2019; 303:55–66. 10.1016/j.jconrel.2019.04.003 30954619

[2] Jebril S.; Khanfir Ben Jenana R.; Dridi C. Green synthesis of silver nanoparticles using Melia azedarach leaf extract and their antifungal activities: In vitro and in vivo. Materials Chemistry and Physics 2020; 248:122898.

[3] Kalimuthu K, Cha BS, Kim S, Park KS. Eco-friendly synthesis and biomedical applications of gold nanoparticles: A review. Microchemical Journal. 2020;152:104296.

[4] Nguyen DH, Lee JS, Park KD, Ching YC, Nguyen XT, Phan VHG, et al Green Silver Nanoparticles Formed by Phyllanthus urinaria, Pouzolzia zeylanica, and Scoparia dulcis Leaf Extracts and the Antifungal Activity. Nanomaterials. 2020;10(3):542. 10.3390/nano10030542 PMC7153602 32192177

[5] Le N.T.T.; Trinh B.T.D.; Nguyen D.H.; Tran L.D.; Luu C.H.; Hoang Thi T.T. The Physicochemical and Antifungal Properties of Eco-friendly Silver Nanoparticles Synthesized by Psidium guajava Leaf Extract in the Comparison With Tamarindus indica. Journal of Cluster Science 2020.

[6] Ahmed RH, Mustafa DE. Green synthesis of silver nanoparticles mediated by traditionally used medicinal plants in Sudan. International Nano Letters. 2019;10(1):1–14.

[7] Koduru J.R.; Kailasa S.K.; Bhamore J.R.; Kim K.H.; Dutta T.; Vellingiri K. Phytochemical-assisted synthetic approaches for silver nanoparticles antimicrobial applications: A review. Adv Colloid Interface Sci 2018; 256:326–339. 10.1016/j.cis.2018.03.001 29549999

[8] Rahimi M.; Noruzi E.B.; Sheykhsaran E.; Ebadi B.; Kariminezhad Z.; Molaparast M.; et al Carbohydrate polymer-based silver nanocomposites: Recent progress in the antimicrobial wound dressings. Carbohydrate polymers 2020; 231:115696 10.1016/j.carbpol.2019.115696 31888835

[9] Mashwani ZU, Khan MA, Khan T, Nadhman A. Applications of plant terpenoids in the synthesis of colloidal silver nanoparticles. Adv Colloid Interface Sci. 2016;234:132–41. 10.1016/j.cis.2016.04.008 27181393

[10] Anjali Das CG, Ganesh Kumar V, Stalin Dhas T, Karthick V, Govindaraju K, Mary Joselin J, et al Antibacterial activity of silver nanoparticles (biosynthesis): A short review on recent advances. Biocatalysis and Agricultural Biotechnology. 2020:101593.

[11] Das P, Karankar VS. New avenues of controlling microbial infections through anti-microbial and anti-biofilm potentials of green mono-and multi-metallic nanoparticles: A review. Journal of Microbiological Methods. 2019;167:105766 10.1016/j.mimet.2019.105766 31706910

[12] Farooqi AA, Khalid S, Tahir F, Sabitaliyevich UY, Yaylim I, Attar R, et al Bitter gourd (Momordica charantia) as a rich source of bioactive components to combat cancer naturally: Are we on the right track to fully unlock its potential as inhibitor of deregulated signaling pathways. Food Chem Toxicol. 2018;119:98–105. 10.1016/j.fct.2018.05.024 29753870

[13] Kubola J, Siriamornpun S. Phenolic contents and antioxidant activities of bitter gourd (Momordica charantia L.) leaf, stem and fruit fraction extracts in vitro. Food Chem. 2008;110(4):881–90. 10.1016/j.foodchem.2008.02.076 26047274

[14] Zhang F, Lin L, Xie J. A mini-review of chemical and biological properties of polysaccharides from Momordica charantia. International journal of biological macromolecules. 2016;92:246–53. 10.1016/j.ijbiomac.2016.06.101 27377459

[15] Chen F, Huang G, Yang Z, Hou Y. Antioxidant activity of Momordica charantia polysaccharide and its derivatives. International journal of biological macromolecules. 2019;138:673–80. 10.1016/j.ijbiomac.2019.07.129 31344411

[16] Sandra M. B, Flavia M. V. F-M, Ricardo de Alvares G, Anna Claudia Saad B, Alda Maria Machado Bueno O, Claudia Cristina Teixeira N. Psidium Guajava (Guava): A Plant of Multipurpose Medicinal Applications. Medicinal & Aromatic Plants. 2012;01(04):1000104.

[17] Díaz-de-Cerio E, Verardo V, Gómez-Caravaca AM, Fernández-Gutiérrez A, Segura-Carretero A. Determination of Polar Compounds in Guava Leaves Infusions and Ultrasound Aqueous Extract by HPLC-ESI-MS. Journal of Chemistry. 2015;2015:1–9.

[18] Díaz-de-Cerio E, Gómez-Caravaca AM, Verardo V, Fernández-Gutiérrez A, Segura-Carretero A. Determination of guava (Psidium guajava L.) leaf phenolic compounds using HPLC-DAD-QTOF-MS. Journal of Functional Foods. 2016;22:376–88.

[19] Bhagyashree SB, Ashwini KJ, Namdev VG, Datta P, Srinivas R, Shankar G, et al Analysis of phytochemical profile and antibiofilm activity of Stem Bark extract of Terminalia Arjuna Wt & Arn against the human pathogen Candida albicans. Journal of Pharmacognosy and Phytochemistry. 2016;5(6):345–56.

[20] Jia S, Shen M, Zhang F, Xie J. Recent Advances in Momordica charantia: Functional Components and Biological Activities. International journal of molecular sciences. 2017;18(12). 10.3390/ijms18122555 PMC5751158 29182587

[21] Chokki M, Cudalbeanu M, Zongo C, Dah-Nouvlessounon D, Ghinea IO, Furdui B, et al Exploring Antioxidant and Enzymes (A-Amylase and B-Glucosidase) Inhibitory Activity of Morinda lucida and Momordica charantia Leaves from Benin. Foods. 2020;9(4). 10.3390/foods9040434 PMC7230926 32260400

[22] Fan M, Kim EK, Choi YJ, Tang Y, Moon SH. The Role of Momordica charantia in Resisting Obesity. Int J Environ Res Public Health. 2019;16(18). 10.3390/ijerph16183251 PMC6765959 31487939

[23] Le Thi P.; Lee Y.; Hoang Thi T.T.; Park K.M.; Park K.D. Catechol-rich gelatin hydrogels in situ hybridizations with silver nanoparticle for enhanced antibacterial activity. Materials Science and Engineering: C. 2018; 92:52–60. 30184778 10.1016/j.msec.2018.06.037

[24] Prakash A, Sharma S, Ahmad N, Ghosh A, Sinha P. Synthesis of AgNps By Bacillus cereus bacteria and their antimicrobial potential. Journal of Biomaterials and Nanobiotechnology. 2011;2(02):155.

[25] Siddhanta S, Barman I, Narayana C. Revealing the trehalose mediated inhibition of protein aggregation through lysozyme-silver nanoparticle interaction. Soft Matter. 2015;11(37):7241–9. 10.1039/c5sm01896j 26271458

[26] Le NTT, Nguyen DH, Nguyen NH, Ching YC, Pham Nguyen DY, Ngo CQ, et al Silver Nanoparticles Ecofriendly Synthesized by Achyranthes aspera and Scoparia dulcis Leaf Broth as an Effective Fungicide. Applied Sciences. 2020;10(7):2505.

[27] Owaid MN. Green synthesis of silver nanoparticles by Pleurotus (oyster mushroom) and their bioactivity: Review. Environmental Nanotechnology, Monitoring & Management. 2019;12:100256.

[28] Kim SW, Jung JH, Lamsal K, Kim YS, Min JS, Lee YS. Antifungal Effects of Silver Nanoparticles (AgNPs) against Various Plant Pathogenic Fungi. Mycobiology. 2012;40(1):53–8. 10.5941/MYCO.2012.40.1.053 22783135 PMC3385153

[29] Stepien G.; Moros M.; Perez-Hernandez M.; Monge M.; Gutierrez L.; Fratila R.M.; et al Effect of Surface Chemistry and Associated Protein Corona on the Long-Term Biodegradation of Iron Oxide Nanoparticles In Vivo. ACS applied materials & interfaces 2018;10:4548–4560 29328627 10.1021/acsami.7b18648

[30] Hoang Thi T.T.; Pilkington E.H.; Nguyen D.H.; Lee J.S.; Park K.D.; Truong N.P. The Importance of Poly(ethylene glycol) Alternatives for Overcoming PEG Immunogenicity in Drug Delivery and Bioconjugation. Polymers (Basel) 2020; 12. 10.3390/polym12020298 PMC7077443 32024289

[31] Medici S.; Peana M.; Nurchi V.M.; Zoroddu M.A. Medical Uses of Silver: History, Myths, and Scientific Evidence. Journal of Medicinal Chemistry 2019; 62:5923–5943. 10.1021/acs.jmedchem.8b01439 30735392

[32] Wang J.L.; Alasonati E.; Tharaud M.; Gelabert A.; Fisicaro P.; Benedetti M.F. Flow and fate of silver nanoparticles in small French catchments under different land-uses: The first one-year study. Water Res 2020; 176:115722 10.1016/j.watres.2020.115722 32247257

[33] Stensberg M.C.; Wei Q.; McLamore E.S.; Porterfield D.M.; Wei A.; Sepulveda M.S. Toxicological studies on silver nanoparticles: challenges and opportunities in assessment, monitoring and imaging. Nanomedicine (Lond) 2011; 6:879–898. 21793678 10.2217/nnm.11.78 PMC3359871

